# The Principles and Practice of Dentistry, Simplified and Condensed

**Published:** 1849-04

**Authors:** 


*The Principles and Practice of Dentistry, Simplified and Condensed, for the use of those who value the health of their mouth, by* R. D. ADDINGTON, A. B., D. D.S. Richmond: 1848, pp. 48.

The above is the title of a pamphlet, very well written, containing a brief outline of the anatomy of the mouth, the pathology and therapeutical indications of the diseases of the teeth, and the manner of replacing the loss of these organs. It constitutes a very excellent popular treatise upon the teeth, and from its brevity, is more likely to be read than it would be, if it were more elaborate.

